# The Impact on Environmental Health from Cemetery Waste in Middle Tennessee

**DOI:** 10.3390/ijerph21030267

**Published:** 2024-02-26

**Authors:** Patrick Richardson, Heather Tillewein, Joao Antonangelo, Daniel Frederick

**Affiliations:** 1 Department of Health and Human Performance, Austin Peay State University, Clarksville, TN 37042, USA; prichardson10@my.apsu.edu; 2 Department of Crop and Soil Sciences, Washington State University, Pullman, WA 99164, USA; joao.antonangelo@wsu.edu; 3 Department of Earth and Environmental Sciences, Austin Peay State University, Clarksville, TN 37042, USA; frederickd@apsu.edu

**Keywords:** cemetery waste, formaldehyde, health, groundwater, arsenic

## Abstract

The burial of caskets with arsenic-treated wood and formaldehyde-based embalming fluids can harm the environment and health. Arsenic (As) can leach into water, affecting aquatic life and the food chain. Formaldehyde can contaminate groundwater, risking drinking water and causing health problems. The purpose of this study was to investigate the prevalence of As and formaldehyde in cemetery plots of different ages. For this, we evaluated whether there is a potential for formaldehyde and As from cemetery caskets to contaminate waterways, which could impact livestock and allow transmission to individuals. There were six soil samples (*n* = 6), collected at 2 m depth, close to the buried caskets, as well as two (*n* = 2) groundwater samples (soil + groundwater) collected from a cemetery in Middle Tennessee. The soil was analyzed by an environmental lab using EPA 8315A for formaldehyde and EPA 3050B for As. All samples were below the limit of detection (<LOD) for As and formaldehyde, except for the 1952 soil sample, which presented 2 mg kg^−1^ of formaldehyde prevalence. We determined that there is a low likelihood of contamination of waterways and transmission to individuals. Future research is needed to investigate earlier dates of cemetery plots to determine if prior embalming practices could still impact present-day health outcomes. Also, current dates of cemeteries should be investigated to determine if there is a prevalence of formaldehyde and As.

## 1. Introduction

### 1.1. Prevalence of Death

According to the United States Census Bureau, there were an estimated 3,417,925 deaths in the United States in 2021 [1]. There was a 19% increase in deaths in the United States due to COVID-19 [1]. The average annual number of deaths has steadily increased year over year [1]. In 2021, the burial rate was 36.6.% in the United States, with an estimated average cost of $7848 for the viewing and burial [2]. The funeral industry generates around USD $16.23 billion in revenue and employs 141,002 individuals [2]. There are an estimated 109,000 registered cemeteries in the United States Geological Survey [3]. This number does not account for hidden graveyards, graveyards yet to be discovered, pet cemeteries, and natural graves created by Native Americans [3]. The Department of Veterans Affairs maintains 155 national cemeteries in 42 states [4].

### 1.2. Changing Embalming Standards

In the Middle Ages, embalming required immersing the body into alcohol and inserting preservative herbs into the body [5]. During the 19th century, different solutions and powders were injected into the human body for preservation [5]. In 1861, during the Civil War Era, modern embalming for funeral purposes was introduced in order to prevent the spread of infection before and after burial [5]. In 1869, August Whilhelm formally discovered formaldehyde; this was the advent of it being popularized in embalming. During this time, concentrations of formaldehyde ranging from 3% to 10% were recommended by various authors. During the Civil War era, Thomas Holmes used a variety of solutions containing arsenic (As), creosote, mercury, turpentine, and alcohol [5]. Another embalmer recommended a 6% solution for the preparation of a body for a funeral, as it avoided browning of the skin (Brenner, 2014). In the 19th and 20th century, formaldehyde was often used for embalming fluid, as individuals did not realize the toxic nature of the chemical [5].

In the mid-19th century, phenol and glycerine solution were introduced into the embalming process [5]. In 1952, embalming fluid was improved by using isopropanol instead of ethanol, sorbitol, and glycerine [5]. In 1966, polyacrylic acid, paradichlorobenzene, and/or orthodichlorobenzene were used in embalming fluids [5]. By 1983, the refrigeration of cadavers became popular, and a what was called a “low formaldehyde” solution was used, but no exact quantity was specified [5]. Today’s embalming solutions contain formaldehyde, phenol, glycerol, and distilled water [6,7]. Embalming fluids currently are exempt from regulation under the Federal Insecticide, Fungicide, and Rodenticide Act [8]. Formaldehyde is replaced in preservation solutions by formalin, which is a diluted version of formaldehyde. By 2007, a range of 4% to 10% formalin was recommended for embalming solutions. By 2012, formaldehyde alternatives began to be considered. Even with alternative options, formaldehyde-based embalming is prone to be favored [5]. About one gallon of embalming fluid is used per 50 pounds for funeral preservation. The goal of embalming is to preserve the body against decomposition [5]. There is little to no research on how the changing standards regarding embalming have impacted decomposition in the body once buried; thus, there is insufficient evidence that the chemical compounds in embalming are not prone to leeching.

### 1.3. Impact of Cemetery Waste

Cemetery waste refers to the minerals, chemicals, and wood composites placed in the ground by way of coffins, vaults, and bodies. These materials break down and leech into the soil and groundwater. The most notable changes in soil composition come from mineral and chemical contamination. Higher concentrations of zinc, nickel, lead, and other potentially toxic metals have been found in gravesite areas as compared to non-burial grounds [9]. This estimate does not account for the other contaminants placed in the ground, such as plastics, varnishes, and embalming fluid. These contain high concentrations of arsenic (As) [9]. According to the Centers for Disease Control and Prevention, exposure to inorganic As can result in skin conditions, increased risk of diabetes, high blood pressure, and several types of cancer [10]. Additionally, embalming fluid contains high rates of formaldehyde, which the Occupational Safety and Health Administration (OSHA) denotes as cancer causing and toxic [10].

Formaldehyde, known to be highly toxic and a carcinogen, is used in various products, such as fertilizers, paper, food preservatives, and medicines, as well as in embalming [11]. Morticians, during the embalming process, are exposed to formaldehyde at nine parts per million (ppm) [11]. There are negative health effects from short-term exposure and long-term exposure during the embalming process. For short-term exposure, embalmers may experience eye, nose, or throat irritation if exposed up to 5 ppm of formaldehyde [11]. An embalmer exposed to 10–20 ppm of formaldehyde may experience cough, chest tightness, and unusual heartbeat [11]. Also, repeated exposure can lead to cancer [11]. For an embalmer exposed to 50–100 ppm of formaldehyde, individuals may experience fluid in the lungs, followed by death [11]. According to OSHA, the permissible exposure limit (PEL) is 0.75 ppm of air measured as an 8 h time-weighted average [12]. The standard is 2 ppm, with a maximum exposure of 15 min [12]. Ingesting as little as 30 mL of a 37 percent solution of formaldehyde (formalin) can result in death, and chronic exposure is linked to cancer [13].

Much of the available literature that exists on cemetery waste examines locations outside of the United States. The studies that do exist were conducted in Michigan and Ohio and only looked at pathogen or mineral contamination of the soil, not chemical contamination [14]. Additionally, there are a few studies from outside of the United states [7]. This research explores if the decomposition of burial-related impacts the soil composition at burial sites [7]. It, however, does not examine the impact that this contamination has on the health of those living adjacent to these areas or the environment as a whole. One article discusses toxic organic volatiles in cemeteries and the threat to human health [15]. The article also mentions the need for research to study leaching from cemeteries and alternative options [15]. Other studies that look at the actual decomposition and purification of human bodies often address the pathogenic risks but do not look at how chemicals like formaldehyde and As are broken down or distributed into the soil and groundwater. Also, previous research does not identify the length of decomposition of the corpse in relation to standards of embalming changing over time.

Further research is needed to address the effects of modern and historic burial. Burial and embalming practices have varied widely through time and by region, which could yield different results. Additionally, soil compositions, climate, and time play influential roles in the breakdown and distribution of contaminants in soil [16]. This calls for regional research to be done in order to better understand the nuances of how burial practices can impact the environment, agriculture, and the health of populations near burial sites. The purpose of this study was to investigate the prevalence of As and formaldehyde in cemetery plots of different ages. The study evaluated whether there is a potential for formaldehyde and As from cemeteries to contaminate waterways, which could potentially impact livestock and the health of individuals.

## 2. Methodology

### 2.1. Overview

Soil analysis in proximity to burial sites is crucial for understanding the impact of human remains on soil properties and biogeochemical processes. To achieve accurate and meaningful results, it is imperative to collect soil samples at a depth that corresponds closely to the vicinity of the interred caskets. The use of ground-penetrating radar (GPR) instruments aids in identifying the spatial distribution of caskets and helps determine the optimal soil depth for sampling. The scientific approach of collecting soil samples at a depth of approximately 2 m, following the GPR-assisted mapping of caskets, ensures that the analyses capture the immediate vicinity of the interred caskets. The depth of a grave can range from 1.5 to 3 m, so the researchers chose 2 m (~6 ft), which is typical for a standard grave [17]. Researchers were also informed by the cemetery that the graves in the sample eras were buried up to 2 m below the soil surface. Also, researchers wanted to collect samples as close to the bottom of the casket as possible due to the leeching of chemicals. This depth selection is justified by its alignment with the known positions of caskets, as found elsewhere in the literature, and its ability to facilitate comprehensive investigations into soil properties, biogeochemical processes, and environmental impacts near burial sites. The results of this study will contribute to a better understanding of soil–casket interactions at the studied Tennessee cemetery and their implications for the surrounding ecosystem.

### 2.2. Sampling Locations

**Figure 1:**
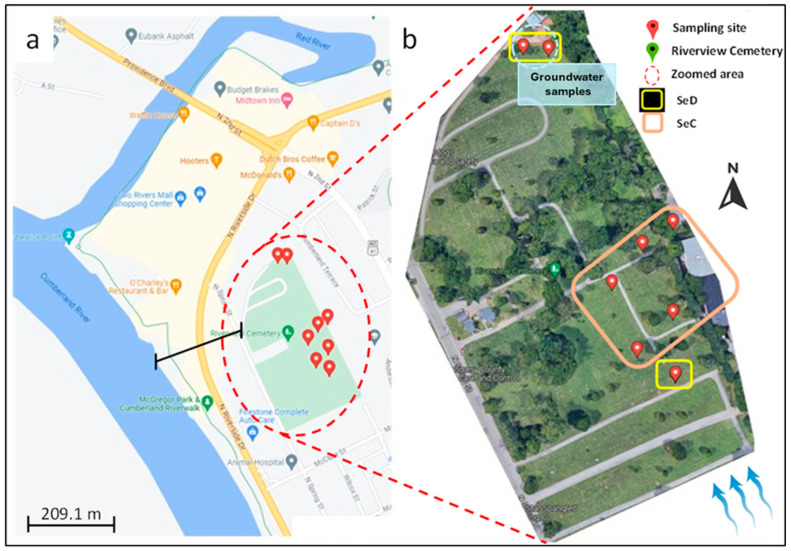
Soil sample locations. (**a**) Cemetery in Middle Tennessee is highlighted in green; both the Cumberland River and the Red River are nearby. Soils were sampled from 6–8′ (72–96” or 183−244 cm) adjacent to or right below the buried caskets, below the B horizon, and closer to the water table. (**b**) Soil sampling sites of the cemetery (zoomed area) and their respective soil series (Web Soil Survey). Blue arrows indicate the direction of the water flow. SeC: Sengtown gravelly silt loam, 5 to 12 percent slopes. SeD: Sengtown gravelly silt loam, 12 to 20 percent slopes. Sengtown series (USDA): https://soilseries.sc.egov.usda.gov/OSD_Docs/S/SENGTOWN.html (accessed on 1 January 2024).

Six soil samples (*n* = 6) were collected at 2 m depth, close to the buried caskets, and two (*n* = 2) groundwater samples (soil + groundwater) were collected, from a cemetery in Middle Tennessee (Figure 1). The two groundwater samples were collected downhill and close to the body of water to which the run-off of soil and groundwater would gravitate (Figure 1). Water flow direction within the landscape was determined through a comprehensive approach, including topographic analysis, field observations, and surface water mapping, after consultation with cemetery representatives. Topographic maps and field observations identified potential flow paths and surface runoff behavior, while surface water mapping delineated water bodies and primary flow directions. This collaborative effort ensured a thorough understanding of water flow dynamics, crucial for assessing contaminant transport pathways to nearby water bodies.

Different eras were selected for soil sampling. This would aid researchers in determining what era had the highest formaldehyde use at embalming, if the concentrations are at levels unsafe for human consumption, and if formaldehyde contamination is moving toward water and at what concentrations. The grave sites selected were determined by previous research on the adoption of formaldehyde in the embalming process and any residual As from burial materials; the years included 1928, 1950, 1952, 1957, 1969, and 1979. The 1928 and 1950 samples were chosen as years before embalmers started using isopropanol instead of ethanol and sorbitol instead of glycerin. The 1952 and 1957 samples represent when embalmers started using isopropanol in embalming. The 1969 and 1979 samples were chosen to assess the effect when embalmers started using polyacrylic acid, paradichlorobenzene, and/or orthodichlorobenzene. The same methods were applied to the secondary samples at the bottom of the cemetery site where groundwater had migrated from the plots (Groundwater Sample A and Groundwater Sample B). This selection was adopted to avoid outliers and obtain a more realistic picture of the entire burial site. After sampling, the preserved soil samples were then transported to a local lab to test for formaldehyde and As. The results were compared to EPA standards for hazardous waste to determine the potential danger to public health.

### 2.3. Soil Properties

The Sengtown series encompasses the characteristics of profound soil depth, effective water drainage, and a moderate level of permeability within upland settings. These inherent soil characteristics facilitate efficient vertical water percolation through the soil profile. Consequently, investigating contaminant dynamics within the Riverview Cemetery environment holds significant scientific relevance. Given the pronounced downward water movement within these soils, understanding the mechanisms and extent of contaminant leaching becomes pivotal in assessing potential environmental risks and formulating effective mitigation strategies.

These soils are formed in residuum weathered from cherty limestone, and the solubility of As in soils from limestone deposits is influenced by factors like limestone composition and pH. Limestone dissolution releases calcium and carbonate ions, raising soil pH. Therefore, higher pH can lead to less soluble As forms by forming precipitated complexes with calcium (Ca), reducing mobility. However, other factors affecting As dynamics must be pointed out, such as mineral interactions, redox conditions, organic matter, and climate. Overall, predicting As solubility requires understanding local conditions through studies and experiments. In that scenario, since the purpose of this work is not to evaluate how different soil properties affect As dynamics, we selected multiple points across the cemetery landscape to enable a comprehensive understanding of As (and formaldehyde) distribution. This approach is effective for land use planning and groundwater protection.

### 2.4. Soil Sampling

Before soil sampling, a GPR survey was conducted to map the spatial location and depth of the caskets within the Tennessee Cemetery. GPR utilizes electromagnetic waves to detect variations in subsurface materials, including caskets, providing a detailed understanding of their positions. Soil was sampled at a depth of approximately 2 m below the soil surface, utilizing a skidder for soil perforation. The chosen soil depth ensured accurate characterization of the soils immediately surrounding and beneath the previously mapped caskets from a cemetery in Middle Tennessee, as determined by GPR instrumentation. This approach guaranteed that the collected soil samples were representative of the burial site environment, enabling comprehensive analyses to be conducted. More precisely, a skid steer, equipped with appropriate perforation tools, was employed to create boreholes at the predetermined sampling locations. Soil samples were then collected from the boreholes. The 2 m depth selection is also justified by its alignment with the known position of caskets, as found elsewhere in the literature, and its ability to facilitate comprehensive investigations into soil properties, biogeochemical processes, and environmental impacts near burial sites to determine formaldehyde and As concentrations.

### 2.5. Soil Analysis

#### 2.5.1. Formaldehyde

Soil samples were properly labeled and homogenized, and around 10–20 g was weighed and transferred into a sample vial. An organic solvent, acetonitrile, was mixed with water and added to the soil. The mixture was shaken using a mechanical shaker and left to stand to facilitate the extraction of formaldehyde from the soil into the solvent phase. The vials were then centrifuged to separate the solvent phase (containing extracted formaldehyde) from the solid soil phase and then transferred to a clean vial for further analysis (EPA 8315A). Derivatization of formaldehyde was carried out when necessary to enhance its detectability and stability. For this, derivatization reagents 2,4-dinitrophenylhydrazine (DNPH) or o-Phthaldialdehyde (OPA) were used. The formaldehyde concentration was determined by High-Performance Liquid Chromatography (HPLC). The HPLC mobile phase was prepared using a mixture of water and acetonitrile, and a buffer was added to control pH and optimize separation. The appropriate HPLC column with suitable stationary phase characteristics was used for the separation of formaldehyde and any derivatized products. A small volume of the extracted and derivatized (if necessary) sample was injected into the HPLC column. Finally, UV–visible spectrophotometer detection at around 360 nm wavelength was performed to quantify formaldehyde by measuring its absorbance. Quality control measures (TNI/NELAC Standards and the laboratory’s Quality Manual) were implemented to ensure the accuracy and reliability of the results.

#### 2.5.2. Arsenic (As)

The As digestion was performed using nitric acid (EPA3050B); 0.5 g of each processed soil sample was predigested for 1 h with 10 mL of trace metal grade HNO_3_ in the HotBlockTM Environmental Express block digester. Digests were then heated to 115 °C for 2 h and diluted with deionized water to 50 mL. Extracts were then filtered through membrane filters of 0.45 µm pore size and determined by an inductively coupled plasma atomic emission spectrometer (ICP-AES) (Pace Laboratories, Madisonville, KY, USA).

#### 2.5.3. Visual MINTEQ Speciation

The distribution of arsenic (As) species across the full pH range (0 to 14) was simulated using Visual MINTEQ version 3.1 [18], a powerful software tool based on a thermodynamic database and capable of predicting speciation and complexation equilibria in aqueous systems. The simulation was conducted by inputting the initial concentrations of 0.01 M arsenate [As(V)], along with relevant physicochemical parameters, such as temperature (25 °C) and default ionic strength (to be calculated). The pH varied systematically from 0 to 14 in increments of 0.1 units. Visual MINTEQ employs a combination of equilibrium chemistry principles and thermodynamic data to calculate the distribution of arsenic (As) species as a function of pH. Species such as H_3_AsO_4_, H_2_AsO_4_^−^, AsO_4_^3−^, and HAsO_4_^2−^ were considered in the model. Further simulation was conducted by introducing calcium ions (Ca^2+^) at an equivalent concentration to ascertain the formation of any corresponding complexes with the precipitate.

## 3. Results and Discussion

The soil analysis of the cemetery plots determined that there was not a prevalence of As or formaldehyde in the soil samples regardless of the date of the casket (plot) (Table 1). The sample from 1952, however, did show a prevalence of formaldehyde at a low concentration of 2 mg kg^−1^. Such a concentration is negligible, preventing determination of whether the formaldehyde was from the actual embalming process or a natural occurrence. The groundwater samples (A and B) were also determined to not have As or formaldehyde present in the samples.

Soil texture, determined by the proportions of sand, silt, and clay, plays a pivotal role in controlling the movement of contaminants. Fine-textured soils rich in clay possess heightened capacities for adsorbing contaminants compared to coarser soils. For instance, As exhibits a strong affinity for clay minerals due to surface complexation reactions [19], while formaldehyde can also undergo adsorption processes with clay minerals [20,21]. Consequently, in clay-rich soils, both contaminants often demonstrate reduced mobility owing to enhanced retention. These observations align with the outcomes of our study, which underscore the minimal presence of such contaminants. Notably, the Sengtown series soils are distinguished by a prominent Argillic horizon, spanning from approximately 11 to 73 inches (~1 to 6 ft) deep (comprising Bt1, Bt2, Bt3, and Bt4 horizons), the precise location of our sampling efforts.

Soil pH affects the speciation of contaminants and the surface charge of soil particles, thereby influencing their mobility. Arsenic, for instance, exhibits different solubility and adsorption behavior under varying pH conditions. At low pH levels, the mobility of arsenic (As) is often heightened due to diminished adsorption onto soil particles [19]. However, it is crucial to note that in acidic conditions, particularly when interacting with certain sorbents, pentavalent arsenic (As(V)) species may not exhibit increased mobility. This phenomenon is attributed to the likelihood of a charge neutralization process occurring, thereby rendering As(V) species immobile. This scenario is particularly probable in soils characterized by a low pH and rich in iron, aluminum, and manganese oxides. The presence of these oxides facilitates the immobilization of As(V) species through complexation and precipitation reactions. Thus, despite the generally observed trend of increased mobility of As at low pH, the specific interactions with sorbents and the composition of the soil matrix play pivotal roles in determining the ultimate fate and transport of As species in acidic environments. Formaldehyde also shows pH-dependent adsorption onto soil minerals, with higher adsorption at lower pH values [20].

**Figure 2:**
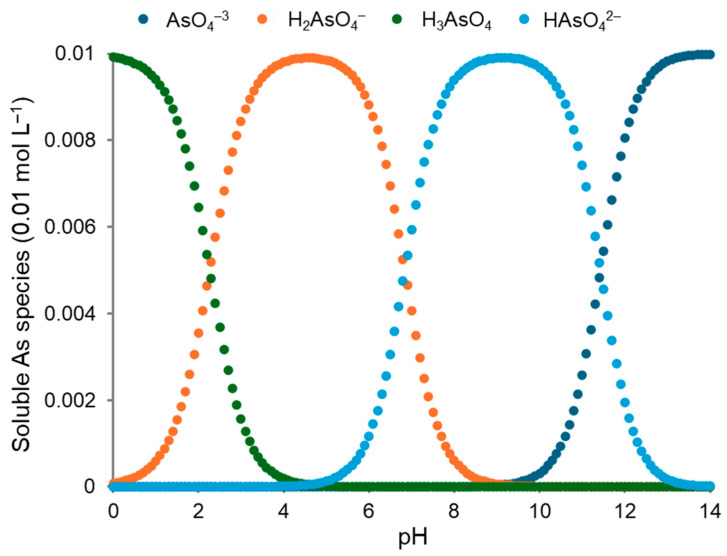
The solubility of arsenate species varies with pH. At low pH (<4), acidic forms like arsenous acid (H_3_AsO_4_) and dihydrogen arsenate (H_2_AsO_4_^−^) are dominant, with low solubility. Near neutral pH (7), hydrogen arsenate (HAsO_4_^2−)^ prevails, showing higher solubility. In highly alkaline conditions (>9), arsenate ions (AsO_4_^3−^) predominate, generally with increased solubility. The figure was generated using data output from Visual MINTEQ software, simulating an As(V) concentration of 0.01 M.

Arsenic is a toxic metalloid and can take on various oxidation states, with arsenate (AsO_4_^3−^) being prevalent in oxygen-rich environments like soil solutions. In this study, arsenate is particularly relevant, since the soil series investigated in the Tennessee cemetery remains aerobic. The solubility of arsenate species varies significantly across different pH levels (Figure 2). At low pH values (pH < 4), As primarily exists in acidic forms such as arsenous acid (H_3_AsO_4_) and dihydrogen arsenate (H_2_AsO_4_^−^), which are relatively insoluble and prone to precipitation (Figure 2). Around neutral pH (pH 7), hydrogen arsenate (HAsO_4_^2−^) becomes predominant, exhibiting higher solubility compared to its acidic counterparts. However, in highly alkaline conditions (pH > 9), arsenate ions (AsO_4_^3−^) dominate in the solution, generally showing increased solubility. This is true when only As species dominate in the solution; however, this is not a real-world scenario, since the soil solution is composed of several other elements that are actively free.

In soils from the Sengtown series, characterized by neutral to high pH levels, the predominant negatively charged species is prone to be the soluble HAsO_4_^2−^ ion (Figure 2). However, in real-world alkaline scenarios, arsenate ions can react with soil metal cations such as calcium (Ca^2+^), abundant in limestone-derived soils, forming insoluble metal arsenates through precipitation reactions (Table 2). This process decreases the concentration of soluble arsenate ions in the soil solution, effectively reducing arsenate solubility. Although EPA 3050B specifies total As, the concentrations might be underestimated due to incomplete digestion and/or matrix effects. Under calcareous soils, Shahbazi and Beheshti [22] reported varying recovery percentages of total As from several extraction methods, including that specified in EPA 3050B. One of the reasons behind these observations relies on the fact that EPA Method 3050B underestimates total As concentrations in soils due to the incomplete dissolution of As species tightly bound within mineral structures or organic complexes.

Soil organic matter serves a pivotal function in binding contaminants through complexation and adsorption mechanisms. Elevated levels of organic matter typically bolster the retention of both As and formaldehyde within soil matrices [23]. Arsenic readily forms complexes with organic matter, diminishing its mobility and furnishing supplementary adsorption sites for formaldehyde [24]. It is noteworthy, however, that organic matter exerts a more profound influence on As mobility than on that of formaldehyde. This is chiefly because organic matter forms robust complexes with As, thereby curbing its mobility and augmenting its retention in soils, as corroborated by Abedin and Mojiri [24]. Despite its capacity to adsorb formaldehyde, organic matter’s impact on the mobility of this compound may be comparatively less pronounced.

The precedence of organic matter in regulating As over formaldehyde mobility can be attributed to the disparate chemical behaviors of these substances in soil. Arsenic readily engages in complexation with organic matter, thereby curtailing its mobility through strong binding with organic ligands. Conversely, although organic matter can also adsorb formaldehyde, the latter may exhibit a diminished affinity for organic matter, owing to disparities in chemical structure and interactions, resulting in a relatively lower influence on its mobility in contrast to As.

pH, ionic strength (*I*), and charge difference were calculated, respectively, as 0.0265 mol L^−1^ (Davies), 8.5, and 9.51%. The table was generated using data output from Visual MINTEQ software, simulating a concentration of 0.01 M for both As and Ca. It should be noted that the difference between the sums of anionic and cationic charge (charge difference) was <10%, as observed by Antonangelo et al. [25]. ^(s)^: solid phase.

There are few studies that examine the decomposition of burial-related materials at burial sites. These studies do not examine the potential of these embalming materials and burial materials to be consumed by living livestock and individuals. The practice of embalming can be dated back to the Middle Ages, where herbs were used [5]. Embalming practices have changed over time; powders and different solutions were used in order to prevent the spread of infection [5]. In 1869, formaldehyde started being used in embalming and continued being used into the 20th century [5]. In 1952, isopropanol was used instead of ethanol, sorbitol, and glycerin, but formaldehyde was still being used. The samples (1928 and 1950) would represent embalming processes before this change. Both samples showed no As or formaldehyde present. The samples from after the change in embalming fluid in 1952 (1952 and 1957) also showed no prevalence of As. As mentioned, the 1952 sample did show a prevalence of formaldehyde of 2 mg kg^−1^, but the amount is not impactful. This phenomenon could be due to environmental factors and not from the actual embalming practice. In 1966, polyacrylic acid, paradichlorobenzene, and/or orthodichlorobenzene started being used in the embalming process with formaldehyde. Our samples from after this change in embalming fluid (1969 and 1979) showed no prevalence of As or formaldehyde.

Formaldehyde and As are both known as highly toxic and carcinogenic substances [10,11]. Arsenic causes an increased risk of diabetes, high blood pressure, and cancer [10,11]. Formaldehyde at various levels can have negative health impacts, such as throat irritation, chest tightness, unusual heartbeat, fluid in the lungs, cancer, and even death [10,11]. Ingestion of formaldehyde can cause death and chronic exposure (United States Department of Labor ND). With the 1952 plot being the only sample to show a low concentration of formaldehyde (2 mg kg^−1^), the overall data show that there is a low likelihood of the potential for embalming materials and burial materials to enter into waterways to contaminate livestock. Also, the study shows the lack of potential of humans to come into contact with formaldehyde and As after the burial process.

The potential risks associated with formaldehyde and As contamination originating from the cemetery evaluated are notably low and pose minimal threats to both human and environmental health. The analyses conducted on cemetery soil and groundwater samples consistently indicated that the concentrations of formaldehyde and As remained below the limit of detection (<LOD), reaffirming the limited impact on surrounding ecosystems and communities. The presence of such contaminants was well below levels of concern, underscoring the responsible management practices employed in cemetery maintenance and burial processes. Consequently, the diligent monitoring and adherence to environmental regulations within cemetery facilities have ensured that any potential risk posed by formaldehyde and As contamination remains insignificant, promoting the overall well-being of both human populations and the environment.

## 4. Limitations

Due to the funding of this study, there was a limited number of samples taken and therefore a limited amount of data collected. Also, the lack of variation of cemetery plot dates adds to a limitation of providing a representative sample. A larger sample size would need to be collected in order to make any definite statements. The samples were taken from one site in middle Tennessee and are not a representation of all burial sites in the state.

The detection of formaldehyde at a prevalence of 2 mg kg^−1^ in the 1952 sample raises queries regarding its origin, whether attributable to environmental factors or embalming procedures. Again, the constraints inherent in the study, characterized by a restricted sample size and minimal variation in cemetery plot dates, underscore the necessity for a more extensive sampling regimen to ascertain conclusive findings. Furthermore, as mentioned, the geographical limitation to a single site in middle Tennessee implies the potential non-generalizability of the observed trends to burial sites statewide.

Finally, the temporal scope of the samples, ranging from 1928 to 1979, invites consideration of the dynamic nature of environmental contaminants over time. Given the substantial timeframe between sample years, it is conceivable that contaminants, including formaldehyde, may have undergone alterations or dissipation due to natural processes such as degradation, transport, dilution, and other environmental mechanisms. Consequently, the observed prevalence of formaldehyde, particularly in the context of the 1952 sample, could reflect the temporal dynamics of contaminant persistence rather than solely originating from contemporary embalming practices or immediate environmental sources. Further investigation, incorporating temporal and spatial dynamics, would be essential to elucidate the fate and persistence of contaminants in the sampled environment.

## 5. Conclusions

The study showed that there was no prevalence of formaldehyde or As in the cemetery plot soil samples and groundwater samples, except for the 1952 samples, which had a low amount of formaldehyde. The study showed that regardless of the changes in embalming practices, there is a low likelihood of risk for transmission of formaldehyde and As to humans from cemetery plots and to groundwater from cemeteries. Future research should be done to determine if earlier dates would show formaldehyde and As after soil analysis. Analyzing earlier dates of cemetery plots could determine if there are potential risks with prior embalming practices. Also, future research focusing on different depths of plots and analyzing waterways near the cemeteries may yield different results. The study identifies that there may be no need for caution with regard to potential contamination of waterways near cemeteries and therefore no concern regarding potential human consumption.

## Figures and Tables

**Table 1:** Values for modified reporting limit, method detection limit, and amount of arsenic (As) and formaldehyde in soil samples. The same values are reported for groundwater samples.

Dates of Samples	As (mg kg^−1^)			Formaldehyde (mg kg^−1^)		
	Modified Reporting Limit	Method Detection Limit	Result	Modified Reporting Limit	Method Detection Limit	Result
1928	8.29	7.05	ND *	2	0.9	ND
1950	8.14	6.91	ND	2	0.9	ND
1952	8.64	7.34	ND	2	0.9	2
1957	8.34	7.09	ND	2	0.9	ND
1969	8.02	6.81	ND	2	0.8	ND
1979	8.53	7.25	ND	2	0.9	ND
Groundwater Sample A	8.08	6.87	ND	2	0.9	ND
Groundwater Sample B	8.00	6.80	ND	2	0.8	ND

* ND, not determined (<LOD).

**Table 2:** Concentrations of aqueous inorganic species and distribution of components between dissolved and precipitated phases.

Component	Species	Total Dissolved		Total Precipitated	
		mol L^−1^	%	mol L^−1^	%
As(V)	AsO_4_^3−^	0.000008	0.08	0.009992	99.92
	H_2_AsO_4_^−^	0.000140	1.40	0.009860	98.60
	H_3_AsO4	0.000000	0.00	0.010000	100.00
	HAsO_4_^2−^	0.007197	71.97	0.002803	28.03
	Total (As)	0.007344	73.44	0.002656	26.56
Ca	Ca^2+^	0.006016	60.16	0.003985	39.85
	CaOH^+^	0.000000	0.00	0.010000	100.00
	Total (Ca)	0.006016	60.16	0.003984	39.84
As(V) + Ca	Ca_3_(AsO_4_)_2_·4H_2_O_(s)_	0.000000	0.00	0.001328	13.28
	Total (As)	0.000000	0.00	0.002656	26.56
	Total (Ca)	0.000000	0.00	0.003984	39.84

## Data Availability

Data will be provided up request from the corresponding author.
